# How to Deal With Post-viral Cutaneous Eruptions in the Era of Coronavirus Infection

**DOI:** 10.3389/fmed.2020.00224

**Published:** 2020-05-12

**Authors:** Alvise Sernicola, Mauro Alaibac

**Affiliations:** Unit of Dermatology, University of Padua, Padua, Italy

**Keywords:** coronavirus, SARS-CoV-2, skin eruption, dermatology, epidemic

In the age of the coronavirus epidemic, the implementation of urgent surveillance measures is sorely needed due to the lack of treatment for coronaviruses and the risk of transmission during the incubation period.

The risk of severe acute respiratory syndrome coronavirus 2 (SARS-CoV-2) transmission is a current topic of investigation and the extent of transmission from individuals who are asymptomatic or within the incubation period remains to be understood (1). The implementation of serologic screening tests is highly anticipated (2) and expected to shed light on the role of asymptomatic infections as well as support the importance of detecting subjects that are asymptomatic or those that have mild symptoms. These individuals remain undetected and complicate the efforts made to control the spread of the disease (3). According to recent reports, up to 51% of confirmed cases may be asymptomatic at the time of diagnosis (4), accounting for silent infection and the incubation period. The latter is reported to last a median of 4 (5) or 5.2 days (6) following infection, with onset of disease within 14 days. How can dermatologists contribute to health surveillance during the coronavirus outbreak?

A recent single center report from Italy provided the first epidemiological data on the skin's involvement in 88 hospitalized patients with COVID-19 (7). Eighteen patients (20.4%), with a positive virology and no history of recent drug intake, developed skin manifestations. Eight patients developed cutaneous involvement at the onset of disease and 10 patients developed cutaneous involvement after hospitalization. Reported skin involvement was consistent with that commonly observed during viral infections and was described as an erythematous rash in 14 patients, a urticarial rash in three patients, and a chickenpox-like vesicular rash in one subject. According to the author, cutaneous signs were not relatable to the severity of the systemic disease.

Shortly thereafter, in a Chinese series of seven critical COVID-19 patients, acral ischemia presenting as cyanosis of fingers and toes with blisters and gangrene was reported in all subjects and correlated to an hypercoagulative state secondary to viral infection (8).

Presently, an unusual occurrence of acral ischemic lesions is being reported in Italy in a growing number of apparently healthy children, adolescents, and young adults. Whether these lesions could be due to virally induced microvascular thrombosis and endothelial damage remains speculative and is supported by positive viral swabs in two cases and by a suspicious family history in the remaining reports (9).

In our routine clinical practice during the COVID-19 outbreak, we are observing a growing number of post viral cutaneous eruptions in apparently healthy individuals in the second or third decade of life that we feel is remarkable compared to the usual local epidemiology of this season. We observed multiple rounded erythematous-violaceous lesions appearing on the dorsal and palmar aspect of the fingers (Figure 1) of adolescent/young patients of both sexes that were asymptomatic or minimally symptomatic for airway disease. We initially interpreted the lesions as an erythema multiforme-like eruption, due to their targetoid shape and peculiar distribution. Considering the latest reports from our country (9), it is reasonable that these acral lesions could be interpreted as signs of acral ischemia secondary to a possible virally induced vasculitis. A Chinese autoptic study provided some preliminary evidence from skin samples of three COVID-19 patients, supporting cutaneous disease involvement (10). Inflammatory damage was reported without evidence of viral epidermal tropism, hinting at an immune-mediated reaction targeting this district that is not related to the presence of SARS-CoV-2 in the skin. A dermatopathologist from our country has shared the report of skin biopsies performed on two patients with COVID-19 disease, matching the histology of Giannotti-Crosti syndrome, that is a non-specific manifestation of a viral infection (11). Due to the unpredictable rate of asymptomatic carriers in this stage it is difficult to speculate on the proportion of subjects with COVID-19-related skin lesions. However, our observations occurred mostly in the month of March and their incidence apparently decreased over April, possibly reflecting the concurrent decline in the transmission of SARS-CoV-2 in our area.

**Figure 1:**
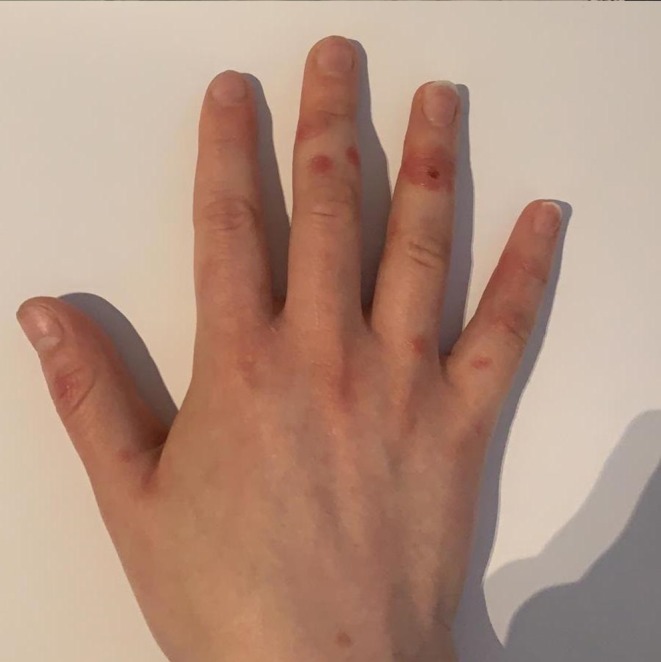
Multiple rounded erythematous-violaceous lesions on the dorsal aspect of the fingers in a 26-year-old female patient with a history of mild respiratory symptoms.

In the current outbreak, and considering the predicted high rate of asymptomatic cases, we wondered if these cases should be tested for the presence of novel SARS-CoV-2, as the serology to commonly associated viral agents is negative. However, due to a current shortage of diagnostic tests related to an ongoing emergency we could not perform a diagnosis of SARS-CoV-2 infection.

It is remarkable to notice that these virally induced immune-mediated skin eruptions apparently occur in patients that have negligible symptoms of a systemic viral disease. These observations hint at the possible role of specific genetic factors that, while a predisposition to the development of skin eruptions, may protect from severely symptomatic presentations of coronavirus infection.

In our current cases of atypical skin eruptions, in which a relationship with conventional viral agents has been ruled out by laboratory testing and clinical history, molecular testing with PCR could be performed on pharynx swabs to support the hypothesis of a possible association with the novel coronavirus. Forthcoming rapid and specific serum assays are expected to aid in the identification of asymptomatic carriers, which is key to controlling the spread of disease. In conclusion, highlighting a relationship between SARS-CoV-2 and virus-induced skin conditions may help identify asymptomatic carriers that are a critical source of inter-human transmission.

## Author Contributions

MA conceived the idea. AS designed and drafted the manuscript. All authors contributed to the writing of the final version of the article.

## Conflict of Interest

The authors declare that the research was conducted in the absence of any commercial or financial relationships that could be construed as a potential conflict of interest.
